# Correlative light and X-ray tomography jointly unveil the critical role of connexin43 channels on inflammation-induced cellular ultrastructural alterations

**DOI:** 10.1016/j.heliyon.2024.e27888

**Published:** 2024-03-21

**Authors:** Chidinma Adanna Okolo, Jack Jonathan Maran, Amy Watts, Jaime Maripillan, Maria Harkiolaki, Agustín D. Martínez, Colin R. Green, Odunayo Omolola Mugisho

**Affiliations:** a Beamline B24, Life Sciences Division, Diamond Light Source, Didcot, Oxfordshire, United Kingdom; b Buchanan Ocular Therapeutics Unit, Department of Ophthalmology, New Zealand National Eye Centre, University of Auckland, New Zealand; c Centro Interdisciplinario de Neurociencias de Valparaíso (CINV), Universidad de Valparaíso, Valparaíso, Chile; d Department of Ophthalmology, University of Auckland, New Zealand

**Keywords:** Connexin43, Hemichannels, Inflammasome, Inflammation, Correlative imaging, Structured illumination microscopy, Soft X-ray tomography, Correlative light and X-ray tomography

## Abstract

Non-junctional connexin43 (Cx43) plasma membrane hemichannels have been implicated in several inflammatory diseases, particularly playing a role in ATP release that triggers activation of the inflammasome. Therapies targeting the blocking of the hemichannels to prevent the pathological release or uptake of ions and signalling molecules through its pores are of therapeutic interest. To date, there is no close-to-native, high-definition documentation of the impact of Cx43 hemichannel-mediated inflammation on cellular ultrastructure, neither is there a robust account of the ultrastructural changes that occur following treatment with selective Cx43 hemichannel blockers such as Xentry-Gap19 (XG19).

A combination of same-sample correlative high-resolution three-dimensional fluorescence microscopy and soft X-ray tomography at cryogenic temperatures, enabled in the identification of novel 3D molecular interactions within the cellular milieu when comparing behaviour in healthy states and during the early onset or late stages under inflammatory conditions. Notably, our findings suggest that XG19 blockage of connexin hemichannels under pro-inflammatory conditions may be crucial in preventing the direct degradation of connexosomes by lysosomes, without affecting connexin protein translation and trafficking. We also delineated fine and gross cellular phenotypes, characteristic of inflammatory insult or road-to-recovery from inflammation, where XG19 could indirectly prevent and reverse inflammatory cytokine-induced mitochondrial swelling and cellular hypertrophy through its action on Cx43 hemichannels. Our findings suggest that XG19 might have prophylactic and therapeutic effects on the inflammatory response, in line with functional studies.

## Highlights

• Cellular hypertrophy (swelling), triggered by pro-inflammatory cytokines, was observed in both parental HeLa cells, and heightened in HeLa cells transfected with connexin43. This swelling was mitigated by the connexin hemichannel blocker Xentry-Gap19 in connexin43 transfected cells only.
• The change in mitochondrial volume following exposure to pro-inflammatory factors occurs independent of Cx43 hemichannel activity, with Cx43 hemichannels further amplifying the mechanism involved in mitochondria swelling.
• Abrupt replacement of pro-inflammatory media with normal growth media further exacerbates the impact of pro-inflammatory cytokines akin to an ischaemia-reperfusion injury. This effect is prevented by Xentry-Gap19 treatment.
• The connexin hemichannel blocker Xentry-Gap19 might have prophylactic and therapeutic effects on the inflammatory response, in line with functional studies.

## Graphical abstract

**Figure:**
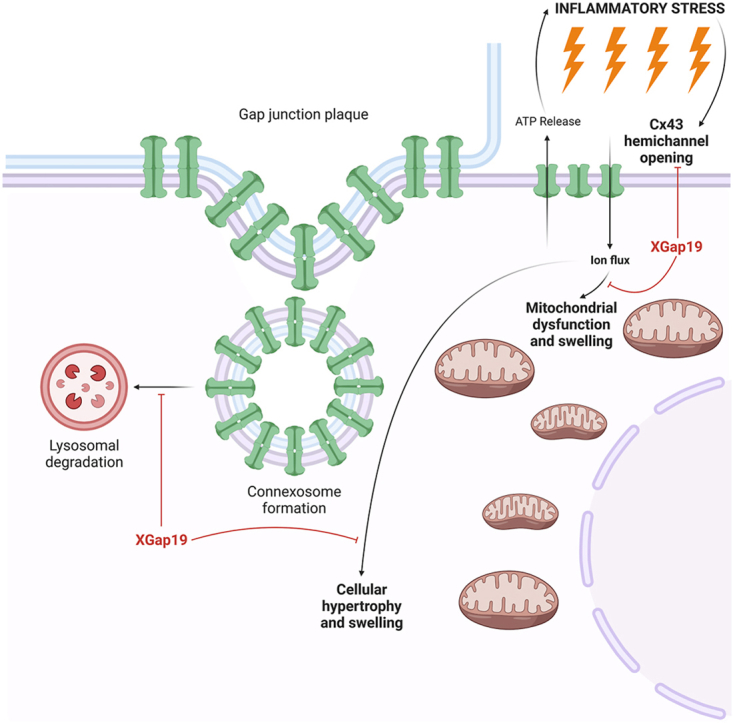

## 1 Introduction

Connexins are a family of proteins with molecular weights ranging from 26 to 70 kDa (kDa) [[1], [2], [3]], with each of the 21 known connexin subtypes named according to its molecular weight in kDa [4,5]. Six connexin protein subunits oligomerize to form a hexamer known as a hemichannel (connexon). Connexins are co-translationally integrated into the endoplasmic reticulum [6] before their assembly into connexons [7,8] which have an extracellular diameter of 5–7 nm [9]. Time-lapse microscopy has revealed that connexons are delivered in vesicular carriers travelling on microtubules from the Golgi to the plasma membrane and that the routing and insertion of connexons can occur in the non-junctional plasma membranes [10] and in appositional junctional plasma membranes [11]. Following the insertion of connexons to non-junctional the plasma membranes, they then move laterally to reach the margins of channel clusters [10], where they dock head-to-head with another connexon from the opposing cell membrane to form gap junctions (GJ) [12]. The GJ channel formed is a double membrane hydrophilic pore that allows direct communication between cells via the passage of ions and small molecules [13]. GJ channels aggregate to form semicrystalline structure (plaques) in appositional membranes that can contain few to many thousand individual GJ channels [[14], [15], [16]]. GJs can couple cells electrically (e.g. in excitatory cells such as cardiac and neuronal cells) and metabolically by enabling the exchange of ions and signalling molecules such as Ca^2+^ between neighbouring cells [17,18]. It has been found that the connexin protein, Cx43, distributes diffusely throughout the cell membrane except in areas of cell-to-cell contact where punctate-resembling junctional plaques were found [19]. Cell pairs with just diffuse labelling were devoid of electrical coupling while cells containing plaques at areas of cell-to-cell contact were electrically coupled. For plaques larger than 0.2 μm, the extent of electrical coupling increased with plaque number and size [19]. Cells with small plaques are not always coupled, suggesting that a minimal plaque size may be required for GJ channels to allow intercellular communication. Dye transfer was also observed only between cells that had plaques [19].

GJs play essential physiological roles in maintaining cellular homeostasis, with loss of GJ function being detrimental as seen in several diseases [[20], [21], [22], [23], [24]]. It has been shown that cells are capable of both cell-to-cell communication via GJs (e.g. Ca^2+^, InsP3-dependent signalling cascade) [25,26] as well as paracrine/autocrine communications (ATP signalling, prostaglandin E2, glutamate, aspartate and ions) via hemichannels [[27], [28], [29]]. However, it has also been shown that in disease and distress states, the scale is tipped towards ATP signalling via hemichannels, suggesting that plasma membrane connexons are not merely precursors for GJs but can act as functional channels as well [[30], [31], [32]]. For instance, it is generally thought that physiologically, GJs are open while hemichannels are closed, while the reverse is true in pathological conditions, which explains the loss of GJ expression and gain in hemichannel activity in disease [33,34]. Recently, studies have indicated that connexin hemichannels are the primary culprits in connexin-channel-associated cellular dysfunction; of particular interest are Cx43 hemichannels, which form “pathological” pores that open during inflammatory events resulting in the unregulated passage of signalling molecules into and out of cells [[35], [36], [37], [38]]. It has been demonstrated that open Cx43 hemichannels release ATP extracellularly, mediating downstream activation of the inflammasome pathway, a part of the innate immune system that results in inflammatory cytokine release and may cause cell death [35,39,40]. A previous study has shown that inhibition of Cx43 hemichannels abrogates oxidative stress and consequent apoptosis in endothelial cells [41]. Another study also demonstrated that inhibiting Cx43 hemichannels using Gap19 mediates protective effects in oxygen-deprived and reoxygenated cells [42]. Gap19 is a peptide that corresponds to the cytoplasmic loop sequence and, by binding to Cx43, prevents the interaction of the cytoplasmic loop with its carboxyl-terminal, thus keeping hemichannels in a closed conformation without affecting gap junction channels [43].

To date, there is no near-physiological comprehensive ultrastructural study that captures the impact of connexin43-hemichannel-driven-inflammation on organelle interactions within the cellular landscape. Also, there is no native-state high-resolution 3D imaging on the response of cellular organelles when treated with Cx43-hemichannel-specific blockers in pro-inflammatory environments. Hence, this study aimed to ascertain how the cellular ultrastructure respond to Cx43 hemichannel blockade using Xentry-Gap19 (XG19) [44] in healthy cells (non-inflammatory) and cells under inflammatory conditions triggered by pro-inflammatory factors. XG19 is Gap19 conjugated to the cell-penetrating peptide, Xentry [45], which binds to the syndecan-4 receptor to facilitate its transmembrane passage [44]. Correlative cryogenic 3D fluorescence and cryogenic soft X-ray tomography at beamline B24 of the UK's National synchrotron (Diamond Light Source) was used to deliver the high resolution cellular imaging necessary to address this knowledge gap.

Soft X-ray tomography at cryogenic temperatures (cryoSXT) involves the three-dimensional (3D) imaging of vitrified biological samples at a close-to-native state by leveraging the natural absorption of soft X-rays by carbon-rich biological features within the so-called ‘water window’ (284 eV–543 eV) energy [46,47]. Imaging cells within the ‘water window’ results in the oxygen-rich medium (composed of water) being relatively transparent. In contrast, the carbon-dense structures absorb and attenuate the X-rays, thus generating contrast. Images of cells are collected in a series of angular projections to form a tilt series which can be reconstructed to a 3D tomogram. The high penetrating depth of X-rays at these energies means that samples up to 10 μm or more thickness can be imaged without the requirement to thin them down (necessary for electron imaging methods), thus, ensuring sample integrity. Likewise, there is no requirement for harsh chemical preservation regimes such as chemical embedding because vitrification, which is used for sample preparation in cryoSXT, is the gold standard for near native sample preservation [[47], [48], [49]]. Vitrification not only ensures the cryopreservation of whole cells at a near-physiological state, it also confers a degree of protection from radiation damage to cells, while also immobilizing them to prevent chemical or mechanical drift during data acquisition. A complementary 3D technique, cryo-structured illumination microscopy (cryoSIM) [47,50], established at beamline B24 further expands the capacity of cryoSXT. CryoSIM is a 3D fluorescence imaging technique which uses fluorophores to localize chemical features, proteins, or organelles of interest in whole vitrified cells. Thus, cryoSIM serves as a disambiguating tool when paired with cryoSXT, which captures the entire cellular milieu in harmony, giving rise to a technique termed, correlative fluorescent light and X-ray tomography (CLXT) [48,[51], [52], [53], [54], [55], [56]].

In this study, we harnessed the powers of these complementary imaging techniques (CLXT) to understand the lone or combined impacts of inflammatory triggers and Cx43 on subcellular dynamics and behaviour. Overall, this study aimed to connect the dots by using ultrastructural indicators and Cx43 states as a compass under inflammatory conditions, thereby documenting the versatility of XG19 in preventing and potentially rescuing the ripple effects engendered by inflammatory stress.

## 2 Results and discussion

### 2.1 Exposure to pro-inflammatory factors for 24 h results in an increase in the lysosomal breakdown of connexin43, which is prevented by XG19 co-treatment

**Fig. 1:**
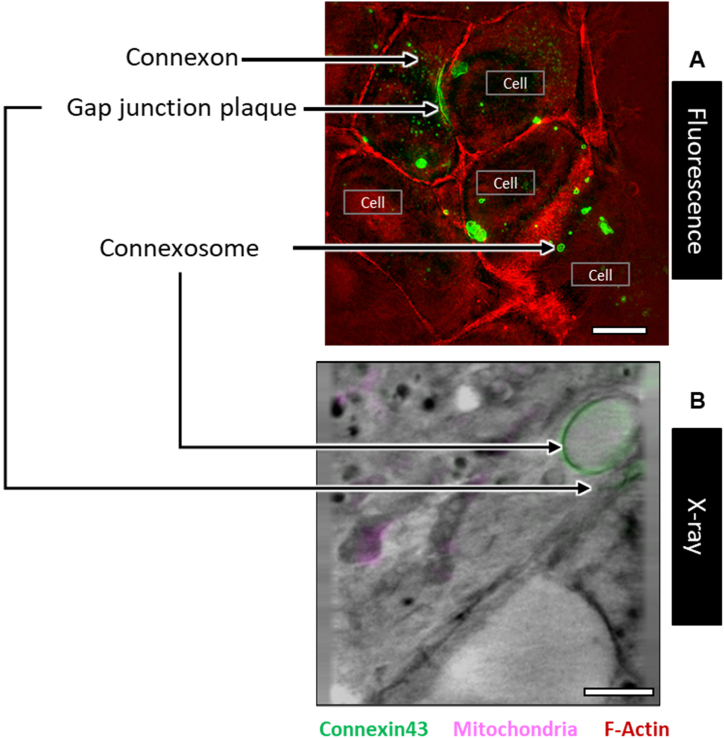
Illustration of connexin43 dynamics in HeLa cells as captured via (A) Fluorescence cryoSIM microscope and (B) Soft X-ray tomography. Green fluorescence represents Cx43-EMD-tagged HeLa cells, red represents fluorescently labelled F-Actin, and magenta represents fluorescently labelled mitochondria. Scale bars (a) 10 μm (b) 2 μm. (For interpretation of the references to colour in this figure legend, the reader is referred to the Web version of this article.)

GJs have a rapid turnover of between one to 5 h [[57], [58], [59], [60]]. Hence, a sole dependence on GJ plaque state and statistics in a non-time-lapsed study such as this, which relied on cryofreezing cells at distinct time points to capture snapshot of events, would not provide us with a holistic picture. Therefore, in order to gain a better understanding of the dynamics of connexin43, HeLa cells were transfected with Cx43-Emerald (Cx43-EMD) and, three states of connexin were examined: connexin43 GJ plaques, annular GJ plaques or close-to-membrane connexosomes [61], and connexons colocalised with lysosomes - an underscore of their degradation fate (Fig. 1) [62]. Cells were outlined using the F-Actin label of the middle slice from cells captured in 3D.

**Fig. 2:**
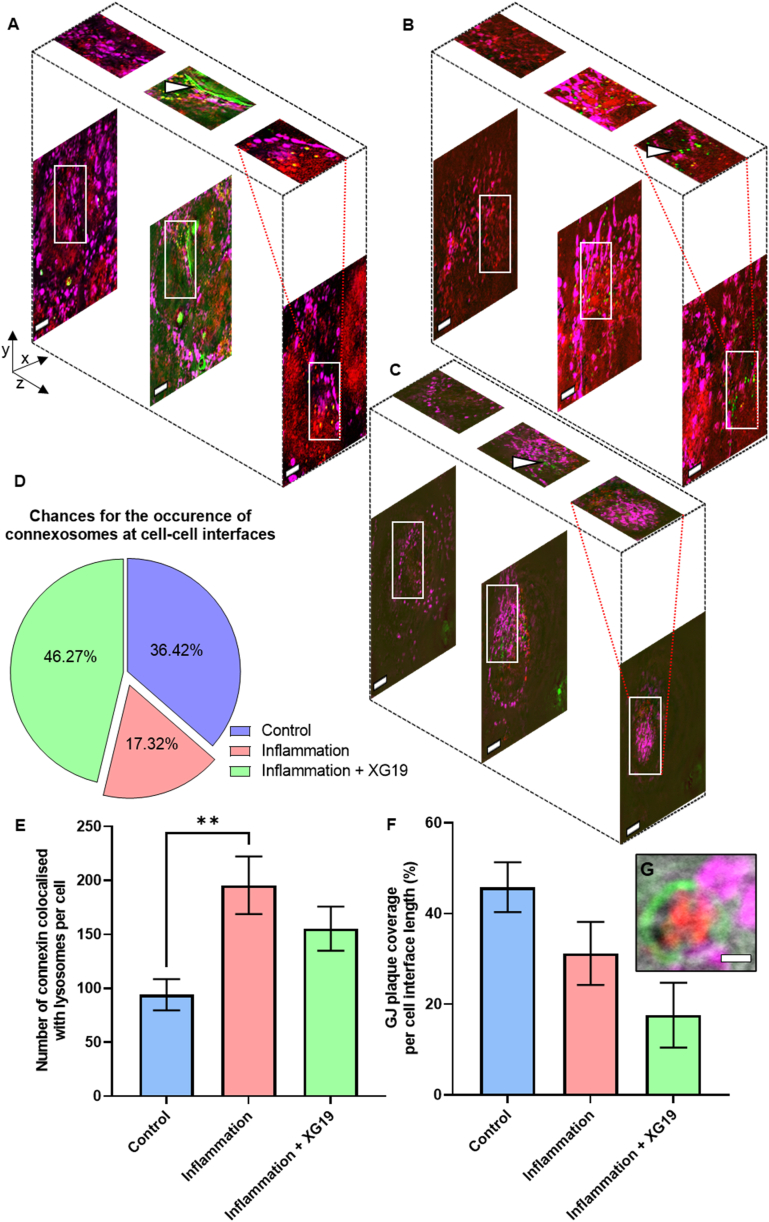
The impact of inflammatory insult on connexin43 channels' fate. Example 3D cryoSIM data from (A) Control Cx43-EMD transfected HeLa cells, (B) Cx43-EMD transfected HeLa cells exposed to pro-inflammatory factors for 24 h, and (C) Cx43-EMD transfected HeLa cells exposed to pro-inflammatory factors and XG19 simultaneously for 24 h. Green fluorescence represents Cx43-EMD-tagged in HeLa cells, red represents fluorescently labelled lysosomes, and magenta represents fluorescently labelled mitochondria. These example images are the bottom, middle and top slices from cryoSIM 3D data showing the distribution of Cx43 as connexons being degraded by lysosomes, hemichannels being transported to the cell membrane or GJ plaques. (D) Chances for the occurrence of connexosomes at cell interfaces. (E) Cx43 co-localisation with lysosome and (F) GJ plaque analyses in control Cx43-EMD transfected HeLa cells (blue bar, n = 18), Cx43-EMD transfected HeLa cells exposed to pro-inflammatory factors for 24 h (pink bar, n = 12) and Cx43-EMD transfected HeLa cells exposed to pro-inflammatory factors and XG19 simultaneously for 24 h (green bar, n = 18). (G) Degradation of connexosomes (green) by lysosomal contents (red). Scale bars = 10 μm. (For interpretation of the references to colour in this figure legend, the reader is referred to the Web version of this article.)

Bottom, middle, and top slices from 3D cryoSIM data show GJ plaques in the middle slices in control cells, akin to the occurrence of plaques at the lateral sides of cell junctions (White arrow in Fig. 2A). To understand the role of Cx43 hemichannels in the ultrastructural rearrangement occurring following pro-inflammatory insult to the cells, XG19 was used to block hemichannels without altering gap junction channels. Previous studies have shown that Gap19 blocks hemichannels in HeLa cells transfected with Cx43 [63].

Slices from 3D cryoSIM data in cells under inflammatory conditions and in XG19 treated cells (Fig. 2B and C) show that GJ plaques appeared less abundant than in control conditions, with connexons observed throughout the volume of the cells. However, quantitative analysis revealed a statistically similar Cx43 GJ plaque coverage at cell interfaces between all groups (Fig. 2F).

$$GJ\;plaque\;coverage\;\left(\%\right)=\frac{GJ\;plaque\;length}{Cell\;interface\;length}\times 100\%$$

Alongside this, a significant increase in the number of connexons colocalised with lysosomes per cell following 24 h of exposure to inflammatory conditions was observed (Fig. 2E). The addition of XG19 simultaneously with the inflammatory trigger showed a subtle decline in the colocalisation of Cx43 within lysosomes, although this failed to reach statistical significance. It is important to note that, even with XG19 treatment, Cx43 localisation within lysosomes after the inflammatory insult was higher than in control conditions (Fig. 2E). Moreover, the chances for peripheral connexosome occurrence decreased during inflammatory stress but increased with XG19 treatment, similar to cells kept in control conditions (Fig. 2D).

These trends may be attributable to a number of reasons. Firstly, heightened proteolytic or lysosomal degradation of connexin has been demonstrated during inflammation, heat stress [64], ischaemia [65], and in failing hearts [66]. Thus, the increase in Cx43 localised in lysosomes under pro-inflammatory factors could be due to an increased lysosomal degradation of Cx43. Hence, given that XGap19-treated cells were treated with pro-inflammatory factors at the same time as XG19, increased degradation of Cx43 during inflammatory stress might be masking the changes brought about following hemichannel blockage with XG19. From a disease perspective, altered and varied expression of Cx43 has been reported in hypertrophic cardiomyocytes [67,68] together with homogenous distribution or heterogenous redistribution of Cx43 [68,69]. Therefore, the remodelling of Cx43 in hypertrophic cardiomyocytes and astrocytes has been a hot topic in this field and findings from our study here shows added insights that increased Cx43 opening will contribute to hypertrophy.

Interestingly, a decrease in peripheral connexosome formation during inflammatory stress that could be reversed by XG19 treatment was observed (Fig. 2D) at the same time as statistically similar levels of Cx43 GJ plaques in all groups (Fig. 2F) (whilst there was a reduction in plaque coverage under pro-inflammatory conditions plus XG-19 treated groups, it was not statistically significant). The failure of XG19 to improve GJ plaque coverage may suggest that XG19's effects are not targeted to GJs and are specific to hemichannels. XG19 is reported to close hemichannels but, conversely, retain the open state of GJ channels, and longer term may enhance GJ coupling [70]. Moreover, this may suggest that GJ derived fragments in XG19-treated cells were probably arrested at a time point in their lifecycle coinciding with the transition between connexosomes to lysosomal degradation, leading to an accumulation of peripheral connexosomes, where the lysosomal breakdown of Cx43 is the rate-limiting step. This theory is supported by our data which showed that lysosomes could directly degrade connexosomes without any intermediary step involving autophagolysosome formation (Fig. 2G) [[71], [72], [73]] and that connexosome degradation by lysosomes may be the rate-limiting step in GJ degradation.

Furthermore, these findings strongly suggest that XG19 does not significantly impair connexosome or GJ formation. An alternative hypothesis is that hemichannel blocking by XG19 may be a signal for the internalisation of that channel. However, additional cell cycle studies are warranted to confirm or refute this hypothesis. Nevertheless, it appears that although Cx43 production is increased during inflammatory insult, treatment with XG19 may modestly reduce connexon breakdown by lysosomes and restore connexosome levels to a semi-inflamed or non-inflamed state, as seen potentially by preventing the transition of connexosomes to lysosomal breakdown. Taken together, these trends highlight the potential role of co-treatment with XG19 during inflammatory injury, in restoring Cx43 dynamics to a more natural non-triggered state.

### 2.2 The addition of pro-inflammatory cytokines over 24 h promotes hypertrophy of HeLa Cx43-transfected cells through Cx43 hemichannels which is prevented by XG19 co-treatment

**Fig. 3:**
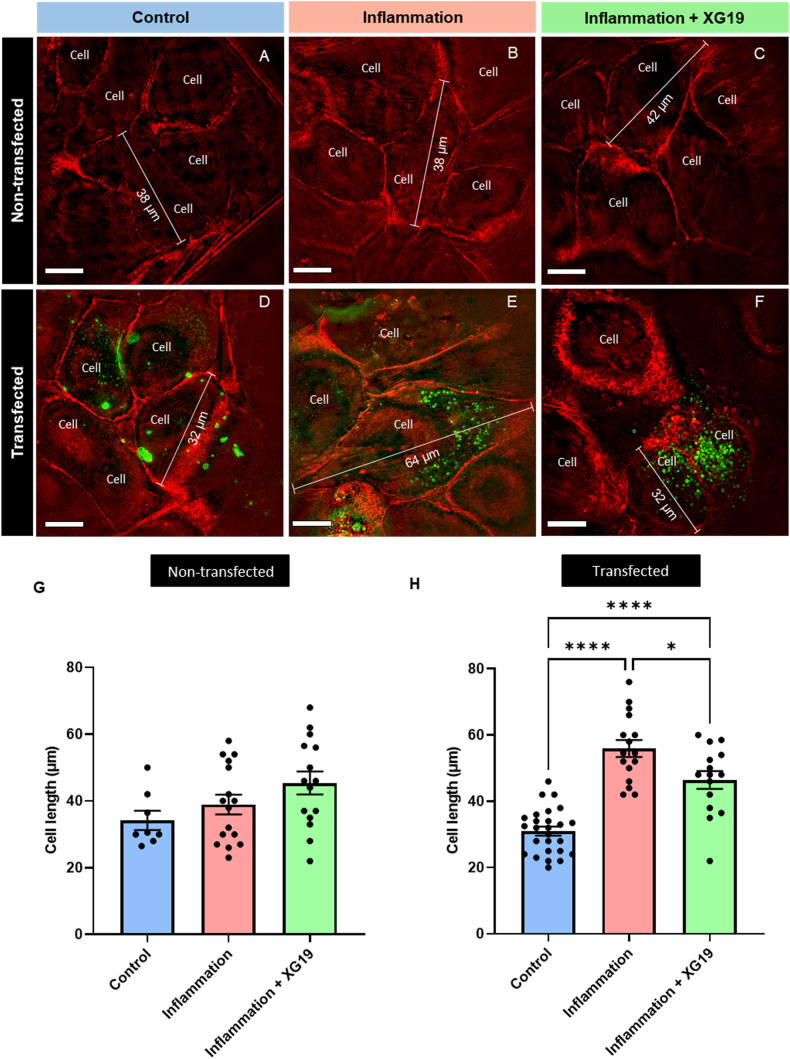
Qualitative representation of cell sizes in (A) parental non-transfected control HeLa cells, (B) parental non-transfected HeLa cells plus 24 h incubation in pro-inflammatory factors, (C) parental non-transfected HeLa cells plus 24 h incubation in pro-inflammatory factors and XG19 simultaneously, (D) Cx43-EMD transfected control HeLa cells (E) Cx43-EMD transfected HeLa cells plus 24 h incubation in pro-inflammatory factors and (F) Cx43-EMD transfected HeLa cells plus 24 h incubation in pro-inflammatory factors and XG19 simultaneously. (G) Quantification of cell length in non-transfected control HeLa cells (blue bar, n = 8), non-transfected HeLa cells at 24 h under pro-inflammatory factors (pink bar, n = 16), and non-transfected HeLa cells at 24 h under pro-inflammatory factors and XG19 simultaneously (green bar, n = 15). (H) Quantification of cell length in Cx43-EMD transfected control HeLa cells (blue bar, n = 26), Cx43-EMD transfected HeLa cells at 24 h of pro-inflammatory condition (pink bar, n = 16), and Cx43-EMD transfected HeLa cells at 24 h under pro-inflammatory condition and XG19 simultaneously (green bar, n = 16) cells. Red represents fluorescently labelled F-Actin, while green represents connexons, connexosomes or GJ plaques [62]. The boundaries of cells were determined using the F-Actin label of the middle slice from cells captured in 3D. Background fluorescence in the green channel was subtracted using the signal in non-transfected cells as the background/autofluorescence level. Scale bars = 10 μm, white measuring bars in A - F represent estimated individual cell length. *p < 0.05, **P < 0.01 and ****P < 0.0001. (For interpretation of the references to colour in this figure legend, the reader is referred to the Web version of this article.)

Non-transfected HeLa cells presented with a similar range of cell sizes regardless of the presence or absence of pro-inflammatory cytokines (Fig. 3A–C). However, we found that the size of Cx-43-EMD transfected HeLa cells increased after 24 h exposure to pro-inflammatory factors compared to control conditions, and this induced cell size increase was prevented by co-treatment with XG19 (Fig. 3D–F and Fig. 3H). On average, two cells were observed per field of view (10 × 10 μm) in inflamed Cx43-transfected HeLa cells, while between four and seven cells were visible in control or XG19-treated cells from a similar 10 μm^2^ field of view. These changes suggest cellular hypertrophy and swelling during exposure to inflammatory conditions in Cx43-EMD transfected cells, which may be prevented by XG19 treatment. In parental non-transfected cells, cell length was statistically similar in all groups, although there was a trend towards increased cell length following inflammatory trigger (Fig. 3A–C and Fig. 3G).

These findings suggest that Cx43 hemichannels activity exacerbates the increase in cell volume triggered by inflammatory signal, allowing a more intense inflammatory responses, which is prevented by co-application of XG19. Although the exact molecular mechanisms underpinning this phenomenon are unclear, previous investigations have suggested that inflammatory conditions promote the formation of pathologically open Cx43 hemichannels, resulting in increased ionic flux, Ca^2+^ and glucose entry, and ATP release into the extracellular space. These changes can result in cellular ionic imbalance leading to fluid (water in media) uptake. ATP-mediated opening of the purinergic receptor (P2X7R) in an autocrine and paracrine manner may also exacerbate ionic fluxes [74]. Inhibition of Cx43 hemichannels by XG19 appears to hinder this process by preventing Cx43 hemichannel opening and ATP release, thereby downregulating both ionic flux and inflammasome activation [74].

From a disease perspective, cellular hypertrophy (swelling) has been previously recorded in Cx43-positive astrocytes of inflammatory demyelinating lesions in multiple sclerosis and allied diseases [75]. The expression levels of Cx43 expression was diminished in such hypertrophic astrocytes as detected with immunohistochemical techniquess [75]. In addition, pro-inflammatory cytokines have been shown to induce marked reduction of astrocytic Cx43 [76]. It is known that in disease, gap junction numbers/coverage often decrease (as shown in Fig. 2F), but that hemichannel opening increases [[77], [78], [79]]. Therefore, with immunohistochemical techniques [75], it is difficult to distinguish between gap junctions and hemichannel clusters, with individual hemichannels falling below the resolution of immunohistochemical techniques. Hence, a decrease in gap junctions (either by immunohistochemistry or total protein assay techniques such as Western blot) does not portray or reflect on hemichannel state or function. Our findings in this present study however underscores the relevance and impact of hemichannels on diseases such as multiple sclerosis [75,78].

### 2.3 Pro-inflammatory cytokine addition for 24 h results in deleterious changes to mitochondria morphology of Cx43-transfected cells, which may be prevented by XG19 co-treatment

**Fig. 4:**
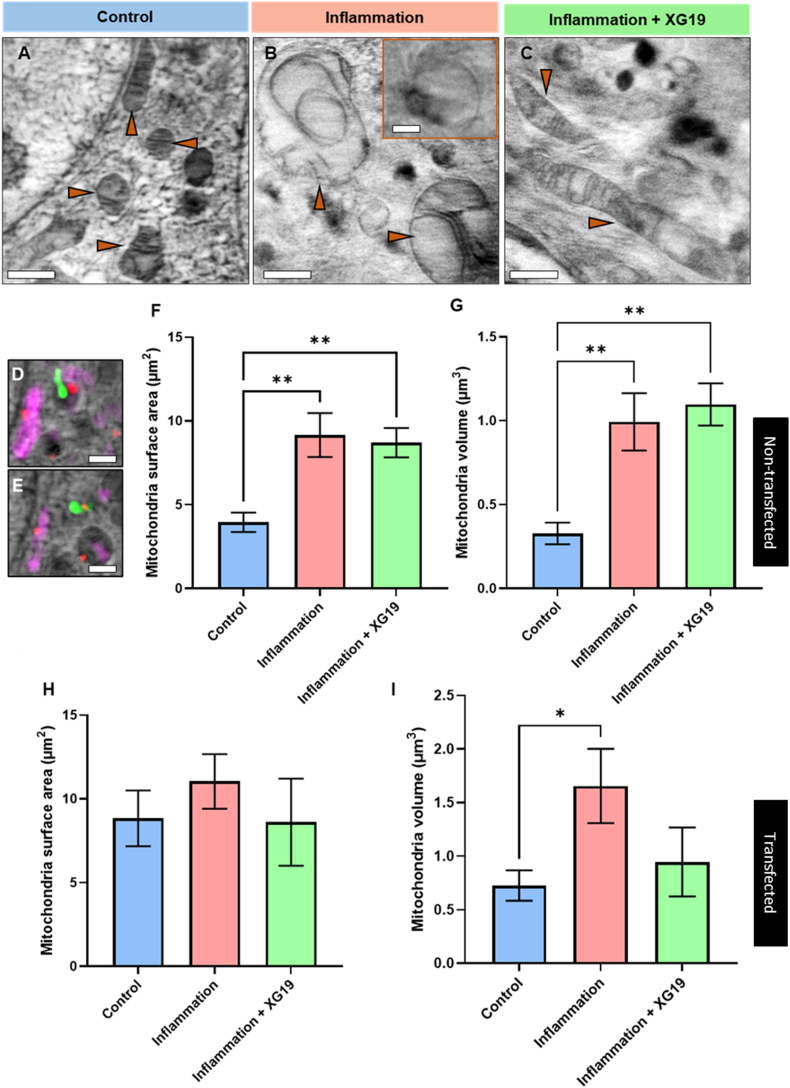
Soft X-ray tomography-guided comparison of mitochondria morphology. Example 5 μm × 5 μm cropped out section of a slice from a 3D tomogram showing morphological variations of mitochondria in: (A) Cx43-EMD transfected control HeLa cells (B) Cx43-EMD transfected HeLa cells at 24 h of pro-inflammatory factors (C) Cx43-EMD transfected HeLa cells at 24 h under pro-inflammatory condition and XG19 simultaneously. (D&E) Ultrastructural interactions at different 3D spaces in the cell showing the degradation of mitochondria by lysosomes. Graphs showing significant differences in (F) mitochondria surface area and (G) mitochondria volume in non-transfected control HeLa cells (blue bar, n = 29), non-transfected HeLa cells at 24 h of pro-inflammatory factor (pink bar, n = 45) and non-transfected HeLa cells at 24 h of pro-inflammatory conditions and XG19 simultaneously (green bar, n = 48). Graphs showing significant differences in (H) mitochondria surface area and (I) mitochondria volume in Cx43-EMD transfected control HeLa cells (blue bar, n = 34), Cx43-EMD transfected HeLa cells at 24 h of inflammatory stress (pink bar, n = 18) and Cx43-EMD transfected HeLa cells at 24 h of inflammatory stress and XG19 simultaneously (green bar, n = 14) cells. Green fluorescence in images represents connexin43-EMD tagged connexons, connexosomes or GJ plaques, red represents fluorescently labelled lysosomes, and magenta represents fluorescently labelled mitochondria. Orange arrows are pointing to mitochondria. Scale bars: (A–C) = 1 μm; inset = 0.25 μm; (D&E) = 0.25 μm *p < 0.05 and **p < 0.01. (For interpretation of the references to colour in this figure legend, the reader is referred to the Web version of this article.)

Representative slices from 3D tomograms demonstrate the states of mitochondria in Cx43 transfected cells under baseline non-inflammatory conditions (Fig. 4A), inflammatory conditions (Fig. 4B) and following XG19 treatment (Fig. 4C). Using this technique of correlative fluorescence and X-ray tomography, we also capture the degradation of mitochondria by lysosomes (Fig. 4D and E), an event which had never been recorded prior to now using a combination of both techniques. An incidental finding from our study was the occurrence of mitochondrial hypertrophy following the addition of pro-inflammatory cytokines to parental HeLa cells in a statistically significant manner (Fig. 3F and G). Exposure to pro-inflammatory factors in the non-transfected parental cells increased mitochondrial surface area and volume, which was not reversed by XG19 co-treatment (Fig. 4F and G). Interestingly, the basal level of mitochondria surface area in control Cx43 transfected cells was already elevated (The blue bar in Fig. 4H).

Although differences in the mitochondrial surface area in Cx43-transfected cells were not statistically significant, inflammatory insult did result in a modest increase in surface area which was reversed by XG19 treatment (Fig. 4H). Again, the basal level of mitochondrial volume in Cx43-transfected cells were already elevated (The blue bar in Fig. 4I). Exposure of these Cx43-transfected cells to pro-inflammatory conditions resulted in a further significant increase in mitochondrial volume. Co-treatment of the pro-inflammatory cytokine-exposed Cx43-transfected cells with XG19 appeared to reduce this increase in mitochondrial volume.

The baseline increase in mitochondria surface area and volume following Cx43 transfection may be attributed to the additional impact of Cx43 expression in transfected cells, leading to higher cross-talk and ion exchange or ATP release to neighbouring cells and the extracellular environment [30]. Cx43 expression may have led to a basal shift in the size and volume of mitochondria to a semi-enlarged state, where both inflammatory insult and Cx43 expression increased mitochondrial size. Notably, these effects occur synergistically in transfected cells exposed to pro-inflammatory factors, significantly increasing mitochondrial volumes to levels much higher than in transfected control cells.

The complete mechanism underlying mitochondrial swelling is presently unclear. However, it is known that calcium levels may rise during metabolic stress or injury, leading to a decrease in the mitochondrial membrane potential and an increase in the permeability of the mitochondrial membrane following the opening of the mitochondrial permeability transition pore (PTP), leading to mitochondrial swelling [80,81]. Furthermore, the opening of the PTP can result in outer mitochondrial membrane rupture and is thought to be vital in leading cells towards some forms of necrotic cell death [81]. Notably, Ott et al. (2007) showed that TNF-α and IL-1 can increase intracellular concentrations of proteins that comprise the membrane transport proteins (MTP), further amplifying this pathway [82]. It is important to note that pathologically open Cx43 hemichannels may result in calcium influx through ATP-mediated P2X7 receptor opening [74]. Furthermore, inflammasome activation from inflammation and extracellular ATP can increase IL-1 levels [74]. These changes during inflammation may synergistically amplify an increase in mitochondrial dysfunction, permeability, swelling and cellular necrosis. XG19 may prevent this by reducing ATP release and P2X7R activation and calcium influx [74]. Accordingly, we found that XG19 diminished the extent of mitochondrial swelling in Cx43-transfected HeLa cells (Fig. 4I).

Furthermore, direct visualisation of Cx43-transfected HeLa cells provides additional insight into structural changes of mitochondria during inflammatory injury. Compared to control conditions, the mitochondria of cells exposed to inflammatory conditions appear to have increased volumes and a smaller crista-to-matrix ratio (Fig. 4A–C). Statistical analyses show that XG19 co-treatment reduced the average mitochondrial volume in Cx43-transfected cells. However, example mitochondria were purposefully selected and shown in Fig. 4C to depict that even in cases where the mitochondria still presented with increased volume following XG19 co-treatment, the mitochondria undertook a more elongated morphology and appear to have a greater crista-to-matrix ratio. These changes with XG19 may have significant functional implications for cell survival. A notable study found that the elongation of mitochondria during cellular stressors such as starvation prevents them from autophagic degradation, increases mitochondrial cristae, increases ATP synthase activity, and maintains ATP production [83]. This may suggest that XG19 may also confer protective effects to cells during inflammatory stress through altering mitochondrial dynamics, although the exact mechanisms underlying this are yet to be elucidated.

As discussed above, pro-inflammatory cytokines lead to inflammasome complex assembly which is activated by Cx43-mediated ATP release. Cx43 hemichannel block prevents inflammasome activation and the release of further inflammatory cytokines [29]. It has been proposed that mitochondrial dysfunction may be an activator of the inflammasome, and that mitochondria may control inflammatory responses [84,85]. Our results here tend to align with this proposition. Based on the findings in this study, it can be inferred that the change in mitochondrial volume occurs independently of Cx43 hemichannel activity, with Cx43 hemichannels further amplifying the mechanism involved in mitochondria swelling.

### 2.4 Acute pro-inflammatory conditions lead to cellular hypertrophy and reduced GJ plaque coverage, which is reversed by acute XG19 co-treatment

**Fig. 5:**
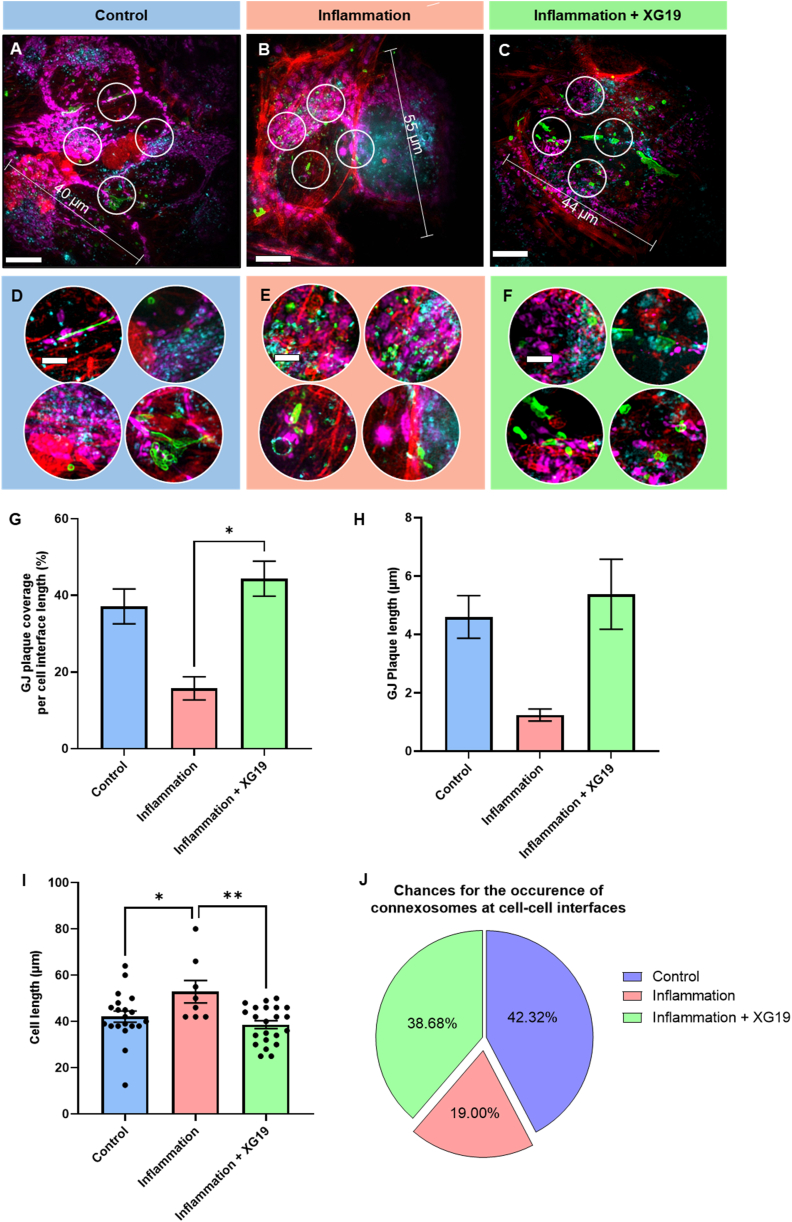
Example slices from cryoSIM fluorescence 3D data showing changes in Cx43 channels' states in (A) control, (B) 1-h inflammatory stress and (C) 2 h inflammatory stress plus 1 h of XG19 co-treatment. (D–F) Insets of different areas in panels A–C highlight the distribution of connexons, GJ plaques or connexosomes. (G) GJ plaque coverage in Cx43-EMD transfected control HeLa cells (blue bar, n = 22), Cx43-EMD transfected HeLa cells vitrified 1 h after inflammatory insult (pink bar, n = 5), and Cx43-EMD transfected HeLa cells vitrified 2-h after inflammatory trigger and 1-h of XG19 co-treatment in the second hour of inflammatory insult (green bar, n = 18). (H) GJ plaque length in Cx43-EMD transfected control HeLa cells (blue bar, n = 28), Cx43-EMD transfected HeLa cells vitrified 1-h after inflammatory insult (pink bar, n = 5), and Cx43-EMD transfected HeLa cells vitrified 2-h after inflammatory trigger and 1-h of XG19 co-treatment in the second hour of inflammatory insult (green bar, n = 39). (I) Cell length of Cx43-EMD transfected control HeLa cells (blue bar, n = 20), Cx43-EMD transfected HeLa cells vitrified 1-h after inflammatory insult (pink bar, n = 8), and Cx43-EMD transfected HeLa cells vitrified 2-h after inflammatory trigger and 1-h of XG19 co-treatment in the second hour of inflammatory insult (green bar, n = 22). (J) Chances for the occurrence of connexosomes at cell interfaces in Cx43_EMD transfected control HeLa cells (blue portion, n = 6), Cx43-EMD transfected HeLa cells vitrified 1-h after inflammatory insult (pink portion, n = 4) and Cx43-EMD transfected HeLa cells vitrified 2-h after inflammatory trigger and 1-h of XG19 co-treatment in the second hour of inflammatory insult (green portion, n = 5). Green fluorescence represents connexin43-EMD tagged connexons, connexosomes or GJ plaques, cyan represents fluorescently labelled lysosomes, red represents fluorescently labelled F-Actin and magenta represents fluorescently labelled mitochondria. Scale bars: (A–C) 10 μm; (insets) 2.5 μm *P < 0.05 and **P < 0.01. (For interpretation of the references to colour in this figure legend, the reader is referred to the Web version of this article.)

Although acute cross-sectional studies provide insight into cellular and molecular changes that may occur during inflammatory stress, it is challenging to ascertain dynamic trends in data without longitudinal data being obtained throughout a cell's life cycle. As such, Cx43 hemichannel states and cellular morphology were examined after 1 h under inflammatory conditions and compared to changes after an additional 1 h under inflammatory conditions (2 h total) with XG19 co-treatment in the second hour of inflammatory insult. Qualitative data from cells analysed longitudinally indicated that Cx43 GJ plaque formation is dramatically reduced during inflammatory stress but is retained or even augmented with XG19 treatment after inflammatory trigger (Fig. 5A–F). This is reflected in quantitative data showing that inflammatory stress resulted in about 50% relative reduction in GJ plaque coverage from control conditions. In contrast, XG19 resulted in a statistically significant increase in the surface area of GJ plaques to levels even greater than control conditions (Fig. 5G). This trend was reflected in GJ length, although without statistical significance (Fig. 5H). These suggest that XG19 indeed does not interfere with connexin synthesis or connexon trafficking. Moreover, as suggested earlier in Fig. 2D and now further corroborated in Fig. 5J, peripheral connexosome occurrence was decreased during inflammatory stress, potentially a knock-on effect from GJ plaques decline, but restored with XG19 treatment (Fig. 2, Fig. 5J). Taken together, these findings emphasise our suggestions from Fig. 2 and further suggest that there may be an increase in connexin overexpression leading to what is seen as an increase in lysosomal degradation of Cx43, and that XG19 likely reverses this by slowing down Cx43 degradation without interfering with Cx43 synthesis. This interpretation gives room for the rationality of our hypothesis that hemichannel activity is increased during inflammation as evidenced by heightened mitochondrial and cellular swelling in Cx43-transfected cells, while also supporting that the heightened lysosomal degradation of Cx43 is due to its heightened production. This most likely reflects changes induced by inflammasome activation, including both the inflammatory response itself and inflammasome mediated epithelial-mesenchymal transition which is also inhibited by Cx43 hemichannel block [86].

Akin to findings in cross-sectional data from Fig. 3, the extent of cellular hypertrophy is outstanding when cell numbers are compared across these conditions across varied time points (Fig. 5I). It is important to state here that the number of images taken is similar (same number of fields of view), and no oversampling has occurred in any experimental group. In the control Cx43 transfected experimental group, nineteen cells were seen in five fields of view, while eight cells were seen in four fields of view in Cx43 transfected HeLa cells vitrified after 1 h under inflammatory conditions. Interestingly, with Cx43 transfected HeLa cells vitrified after 2 h under inflammatory conditions and 1 h of XG19 co-treatment in the second hour after inflammatory trigger, twenty-two cells were seen in five fields of view, showing a return to the control state.

The finding of rapid increase in cell sizes (swelling) 1 h after pro-inflammatory treatment could imply that swelling occurs before inflammasome activation, and that cell swelling is consistent with previous findings that “identify cell volume regulation as a basic conserved homeostatic mechanism associated with the formation of the NLRP3 (nucleotide-binding domain, leucine-rich–containing family, pyrin domain–containing-3) inflammasome and reveal a mechanism for NLRP3 inflammasome activation” [87]. In addition, the observation (Fig. 5I) shows that XG19 not only prevents (cellular swelling as previously shown in Fig. 3H, but also rescues swollen cells to close-to control cell sizes by interfering with hemichannels without affecting GJ formation.

### 2.5 Discontinuation of prolonged exposure to pro-inflammatory factors amplifies cell hypertrophy, while treatment with XG19 for 24 h following discontinuation of pro-inflammatory trigger fuels recovery in Cx43-transfected cells

Cell boundaries were determined using F-Actin fluorescence label at the edges of the cells. 3D X-ray tomograms were used to illustrate cellular ultrastructural changes. In this section, cell width was measured as an indicator for hypertrophy instead of cell length because cells were massively rounded when pro-inflammatory factors were discontinued. This observation is corroborated by the significantly lower aspect ratio (height-to-width ratio) in cells grown for 24 h in pro-inflammatory media plus an additional 24 h without pro-inflammatory media when compared to cells grown in pro-inflammatory media but treated with XG19 after the first 24 h of insult (Figs. S1A–C). Thus, if the cell lengths were measured as had been done in previous analyses before this section, the extent of roundedness and swelling in this condition would have been downplayed.

**Fig. 6:**
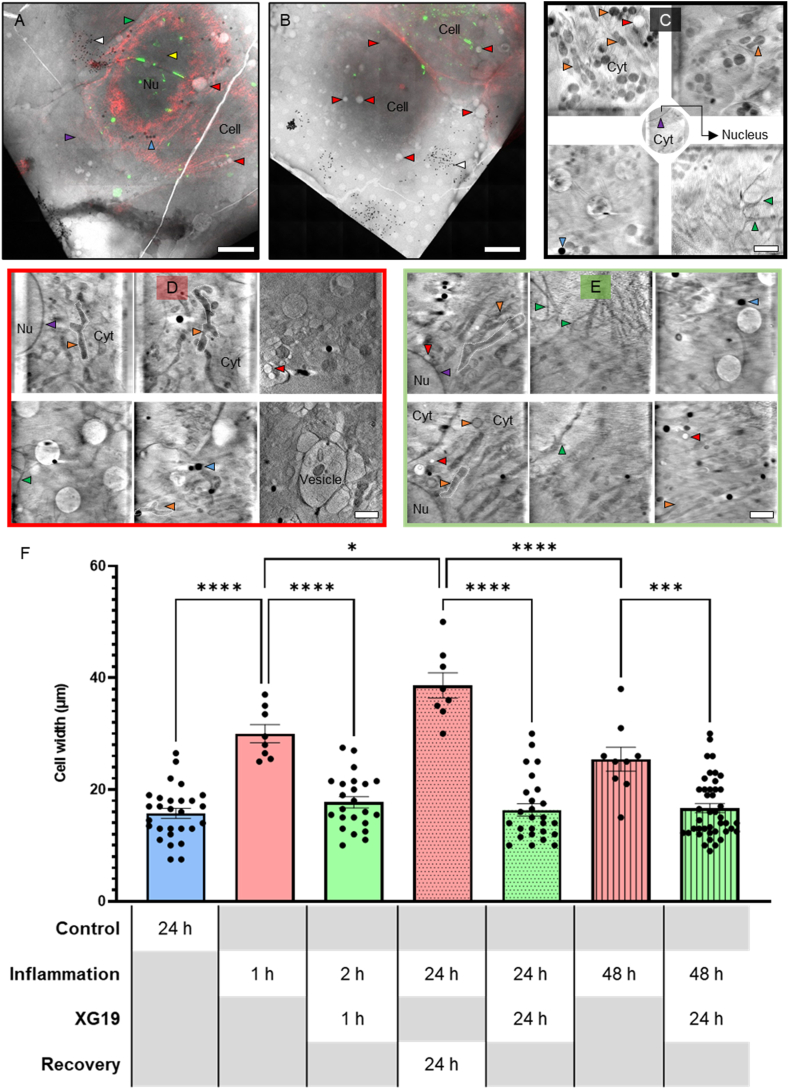
Cellular state and gross morphology. (A) 2D X-ray mosaic showing HeLa Cx43-EMD transfected control cells, (B) 2D X-ray mosaic showing HeLa Cx43-EMD transfected cells at 1 h of inflammatory insult, (C) Slices from 3D X-ray tomograms showing HeLa Cx43-EMD transfected cells at 2 h of inflammatory insult and 1 h of XG19 co-treatment in the second hour of inflammatory injury, (D) Slices from of a 3D X-ray tomogram showing branched mitochondria morphology in cells where pro-inflammatory factors were discontinued for 24 h in HeLa Cx43-EMD transfected cells, (E) Slice of a 3D X-ray tomogram showing elongated mitochondria morphology from cells where pro-inflammatory factors were discontinued for 24 h and treated with XG19 for an additional 24 h in HeLa Cx43-EMD transfected cells. (F) Analyses of gross cell morphology showing cell width in control Cx43-EMD transfected HeLa cells (blue bar, n = 28), Cx43-EMD transfected HeLa cells at 1-h pro-inflammatory trigger (pink bar, n = 8), Cx43-EMD transfected HeLa cells at 2-h of pro-inflammatory factors plus 1 h of XG19 co-treatment in the second hour of inflammatory insult (green bar, n = 23), Cx43-EMD transfected HeLa cells at 24 h of inflammatory condition + pro-inflammatory cytokines discontinued for an additional 24 h (pink dotted bar, n = 8), Cx43-EMD transfected HeLa cells at 24 h of inflammatory insult + pro-inflammatory factors discontinued for 24 h with 24 h of XG19 treatment immediately upon discontinuation of pro-inflammatory factors (green dotted bar, n = 26), Cx43-EMD transfected HeLa cells at 48 h of inflammatory condition (pink striped bar, n = 9), Cx43-EMD transfected HeLa cells at 48 h of pro-inflammatory factors & 24 h of XG19 co-treatment in the last 24 h of exposure to pro-inflammatory factors (green striped bar, n = 42). Nu = nucleus, Cyt = cytoplasm, filopodia (green arrows), nuclear membrane (purple arrows), nucleoli (yellow arrows), vacuoles/vesicles (red arrows), lipid droplets (blue arrows), mitochondria (orange arrows) 200 nm Au nanoparticles (white arrows). Red fluorescence represents F-Actin labelling and green fluorescence denote connexin43-EMD tagged connexons, connexosomes or GJ plaques. Scale bars (A&B) 10 μm, (C–E) 2 μm *p < 0.05, ***p < 0.001 and ****p < 0.0001. (For interpretation of the references to colour in this figure legend, the reader is referred to the Web version of this article.)

As mentioned in the comparison of cell numbers above, the same rationale applies here where a smaller number of cells are recorded following acute (1 h) or chronic (24–48 h) exposure to pro-inflammatory factors when the number of fields of view (images acquired) is similar (Fig. 6A–E). These findings are reflected in quantitative analysis showing that cell width is increased in HeLa Cx43-transfected cells following acute (1 h) and chronic (24–48 h) inflammatory stress (All pink bars in Fig. 6F). Treatment with XG19 appears to promptly rescue cells from swelling, with statistically significant reductions in cell width across all conditions (All green bars in Fig. 6F).

Interestingly, the abrupt replacement of the growth media containing pro-inflammatory factors with normal growth media after 24 h further exacerbated cellular hypertrophy (Fig. 6D and the pink dotted bar in Fig. 6F) when compared to when these cells were exposed to 48 h of constant inflammatory conditions. These morphological changes indicate even more severe cell injury, akin to previous findings in ischaemia-reperfusion models, which showed that abrupt reperfusion with oxygen and nutrients after ischaemia accelerates Cx43 degradation to a greater extent than ischaemia alone [65]. Cx43 hemichannel inhibition by XG19 appears to be effective in preventing this exacerbated inflammatory stress caused by abrupt growth media replacement, similar to previous findings which have shown that XG19 can offer protective effects in hypoxic/reoxygenated and ischaemia-reperfusion injury [42,63,88].

Additionally, nuclear membrane breakdown and vacuole or vesicle formation were observed under acute pro-inflammatory conditions in HeLa Cx43-EMD transfected cells, which are processes associated with cell death [89] (Fig. 6B). Fewer observable vacuoles/vesicles and the presence of nuclear membranes and nucleolus in cells co-treated with XG19 after 1 h of pro-inflammatory conditions (Fig. 6C) suggest that XG19 may cushion against cell death during inflammatory stress. Cells which were left to recover for 24 h following 24 h of exposure to pro-inflammatory factors (Fig. 6D) showed no obvious damage to nuclear compartments, little observable vacuolation and intact cell membranes, thus, distinguishing them from cells exposed to pro-inflammatory factors for 1 h. This could be because of the 24 h recovery period or even because by 24 h, the most adversely affected cells had died, leaving cells which were at steady-state or were still adapting to their current environment. This may also reflect variability in Cx43 expression, and therefore hemichannel numbers, following Cx43-EMD transfection within individual cells.

Moreover, treatment with XG19 following exposure to 24 h of inflammatory conditions appears to give mitochondria a more elongated morphology orange arrows in (orange arrows in Fig. 6E) as opposed to the branched morphology that is observed in cells exposed to 24 h of pro-inflammatory conditions and left to recover without XG19 treatment (orange arrows in Fig. 6D). This adaptation to an elongated mitochondria in Fig. 6E morphology is similar to the adaptation seen in Fig. 4C following XG19 co-treatment during chronic exposure to pro-inflammatory factors. These structural differences have functional implications, where thicker and elongated mitochondria have more space for cristae [90] and are, hence, more able to function efficiently to produce ATP in cells. Therefore, these findings suggest that XG19 may also indirectly drive morphological changes in mitochondria that can optimise function and sustain cell survival amid inflammatory insult via the blocking of Cx43 hemichannels. This also shows that XG19 not only is key in facilitating cell size reduction by regulating the gating of Cx43 hemichannels, but XG19 appears to influence the fine ultrastructural adaptations in these cells prominently. Based on the findings in this study, it can be inferred that the change in mitochondrial volume occurs independently of hemichannel activity, with Cx43 hemichannels amplifying the mechanism involved in mitochondria swelling.

## 3 Conclusion

Correlative light and X-ray tomography has aided the findings of this study. Routine cell biology studies involving the investigation of Cx43 are carried out using immunofluorescence (which involves harsh chemical fixation and permeabilisation of cell membranes), Western blotting, quantitative RT-PCR and other molecular techniques. For the first time, we present a novel approach to deploy cutting-edge synchrotron-based 3D imaging techniques paired with high resolution near-native fluorescence imaging to study the ultrastructure of Cx43 upon pro-inflammatory trigger. We acknowledge that these sophisticated techniques are hardly available in clinical settings, however, we posit that the findings from this study better align us to discern Cx43 degradation fate for the design of targeted therapies. Furthermore, we place the applicability of this technique within the realms of (a) pre-clinical trials for drug design and dosage optimisation, (b) augmenting patient studies due to its high throughput nature to screen for drug effects at high resolution and in the near-native state of the cells and (c) post-market surveillance for adverse drug reactions.

It is generally believed that inflammation leads to decreased gap junction and increased hemichannel expression and opening at the cell membrane [67,68,75,76]. We have demonstrated here that Cx43 degradation is heightened in inflammation. We have also shown that connexosome can be directly degraded by lysosomes without the autophagolysosomal degradation step always necessary. Findings from this present study suggest that the inhibition of Cx43 hemichannels by XG19 may modestly reduce lysosomal breakdown of GJ plaques under inflammatory conditions by potentially preventing the direct degradation of connexosomes by lysosomes. Furthermore, XG19 treatment appears to reverse cellular hypertrophy and dysfunctional changes in mitochondrial structure induced by acute and chronic inflammatory conditions. These findings provide novel insight into the underlying effects of XG19 on Cx43 dynamics and its role in both preventing and reversing inflammatory cytokine-induced cell damage. Importantly, the data suggests that the change in mitochondrial volume occurs independently of hemichannel activity, with Cx43 hemichannels further amplifying the mechanism involved in mitochondria swelling. Further investigations are warranted to fully elucidate the exact molecular mechanisms underpinning the action of XG19 on modulating connexosome breakdown by lysosomes, mitochondria morphology and cellular hypertrophy. Cx43 channels (gap junctions and hemichannels) are the most expressed in the body and most studied in relation to inflammatory conditions. HeLa cells were chosen for use in this study because they do not express connexins to any significant degree. It is noteworthy that there have been reports that HeLa cells might express very low levels of Cx45, a connexin isotype with poor conductance [91]. We therefore chose to overexpress Cx43 in HeLa cells, not to mimic physiological conditions, but to hone in on how channels formed by Cx43 respond to pro-inflammatory insult and drug treatment. It is hoped that a better understanding of how Cx43 channels behave would inform drug development in the future.

While the manuscript may not have direct physiological or clinical applications, it extends our knowledge of how Cx43 behaves following pro-inflammatory insult. A better understanding of the Cx43 degradation mechanisms and channels' states in response to inflammation as well as XG19's pharmacodynamics would equip us to better design or tailor therapeutics. Furthermore, novel findings such as the effect of inflammation and Cx43 hemichannel blockade on the mitochondria reinforce the need to investigate how mitochondrial stress might relate to Cx43 hemichannels.

## 4 Star methods

**Table:** Key Resources Table

REAGENT OR RESOURCE	SOURCE	IDENTIFIER
**Chemicals, peptides, and Recombinant Proteins**		
Dulbecco's Modified Eagle Medium (DMEM)	Fisher Scientific	Cat#11500416
Foetal Bovine Serum (FBS)	Fisher Scientific	Cat# 11550356
Trypsin/EDTA	Fisher Scientific	Cat#11560626
Hanks' Balanced Salt Solution (HBSS)	Fisher Scientific	Cat#12549069
Penicillin/Streptomycin	Fisher Scientific	Cat#11548876
l-Glutamine	Fisher Scientific	Cat#11510626
Sodium Pyruvate	Fisher Scientific	Cat#11530396
Mitotracker Deep Red	Fisher Scientific	Cat#12010156
Lysotracker Blue	Fisher Scientific	Cat#12080146
F-Actin Orange	Fisher Scientific	Cat#17108568
Hoest 33342 NucBlue Live Ready Probe Blue	Fisher Scientific	Cat#12303553
Xentry Gap19	ChinaPeptide Co. Ltd	lclrpvGGKQIEIKKFK (95% purity)
Human TNF-alpha Recombinant Protein	Fisher Scientific	Cat#10056073
Human IL-1 beta Recombinant Protein	Fisher Scientific	Cat# 10394403
Glucose Solution	Fisher Scientific	Cat#15384895
Lipofectamine 2000	Fisher Scientific	Cat# 11513492
Opti-MEM Reduced Serum Media	Fisher Scientific	Cat#11524456
**Experimental Models: Cell Lines**		
HeLa cells	ATCC, Manassas, Virginia, USA	CCL-2
**Recombinant DNA**		
hCx43 mEmerald-N1 plasmid	Professor Viviana Berthoud, The University of Chicago	
**Software and Algorithms**		
SoftWoRX 6.5.2	GE Healthcare	N/A
Chromagnon	Matsuda et al., 2018 [92]	https://github.com/macronucleus/chromagnon
IMOD package (version 4.9.2)	Kremer et al., 1996 [93]	https://bio3d.colorado.edu/imod/
Fiji	Schindelin et al., 2012 [94]	https://imagej.net/Fiji
eC-CLEM	Paul-Gilloteaux et al., 2017 [95]	http://icy.bioimageanalysis.org/plugin/ec-CLEM
Prism	GraphPad	https://www.graphpad.com
**Reagent or Resource**		
Cockpit	Micron, University of Oxford	https://github.com/MicronOxford/cockpit
Linkam's LINK software	Linkam Scientific	https://www.linkam.co.uk/link-controlsoftware
**Other**		
TEM grids	Quantifoil	Cat#AU G200F1 finder
Cryo TEM Grid boxes	Agar Scientific	Cat#AG160-40W
250 nm gold nanoparticle fiducials	BBI Solutions	Cat#SKU EM.GC250
CryoSIM	Beamline B24	Kounatidis et al., 2020 [47] Phillips et al., 2020 [50]
Transmission X-ray Microscope	Carl Zeiss X-ray Microscopy, Inc.	Cat#UltraXRM-S220C
CryoSIM cameras	Oxford Instruments	Cat#Andor iXon Ultra 897
CryoSIM & Axioimager Linkam cryostages	Linkam Scientific	Cat#CMS196 M LED Cryo Correlative Stage
Axioimager microscope	Carl Zeiss AG	Model#Axio Imager 2
Axioimager objectives	Carl Zeiss AG	Model#50x/0,55 DIC
TXRM photon detector	Princeton Instruments	Cat#Pixis1024B CCD
TXRM camera	Teledyne	Cat#Retiga 4000R

## 5 Resource availability

### 5.1 Lead Contact

Further information and requests for resources and reagents should be directed to and will be fulfilled by the Lead Contact, Dr Chidinma Adanna Okolo (Chidinma.okolo@diamond.ac.uk).

### 5.2 Materials availability

This study did not generate new unique reagents.

## 6 Experimental model and subject details

### 6.1 Workflow and experimental design

**Fig. 7:**
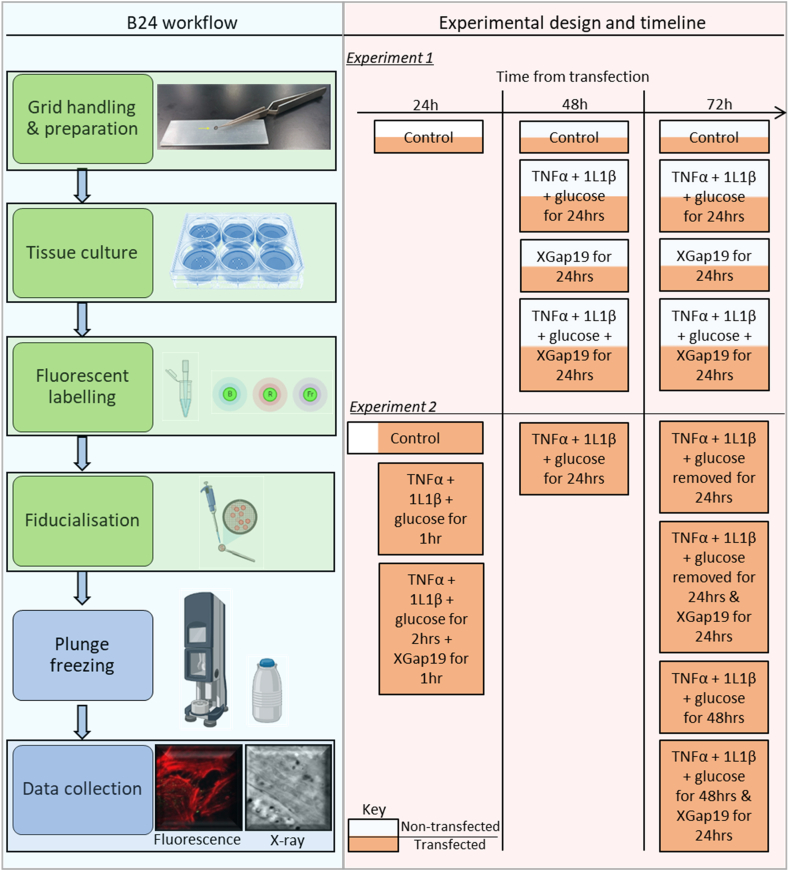
Experimental workflow at beamline B24 together with a flowchart describing the adopted experimental design and timeline.

This study followed the established workflow at beamline B24, Diamond Light Source. First, HeLa cells were cultured and seeded onto hydrophilised transmission electron microscope (TEM) grids. Grids were split into different experimental groups (Fig. 7) and treated accordingly. One hour prior to plunge freezing, fluorescent dyes were added to the live cells to label the desired organelles. Monodispersed gold nanoparticles were then added to fluorescently labelled live cells immediately before the vitrification of the cells via plunge freezing in liquid nitrogen-cooled liquid ethane. Vitrified samples were stored within grid boxes in liquid nitrogen until they were needed for imaging.

The cryo-structured illumination microscope (cryoSIM) at B24 was used for sample mapping and three-dimensional (3D) imaging of fluorescence distribution and localisation in vitrified cells. Cells imaged with the cryoSIM were subsequently transferred to the transmission X-ray microscope for cryo soft X-ray tomography (cryoSXT) of the entire cell volume to document the ultrastructural landscape. Datasets obtained via cryoSIM and cryoSXT were processed and reconstructed, and same-region datasets from both microscopes were correlated with high precision to generate a comprehensive three-dimensional view of regions of interest. Finally, morphometric analyses were carried out using cryoSIM and/or X-ray data.

### 6.2 Grid handling

The established support platform for cell culture at beamline B24 for the purpose of soft X-ray tomography is the 3.05 mm 10.13039/501100001838TEM carbon-coated holey gold grids (Quantifoil, product code: AU G200F1 finder). These TEM grids were always carefully handled and sterilised in 70% (vol/vol) ethanol before use. Excess ethanol was rinsed off in HBSS (Fisher Scientific), and the grids were hydrophilised by submerging them in 6-well cell culture plates containing foetal bovine serum (FBS; Fisher Scientific) for 8–24 h before cells were seed on the grids.

### 6.3 Cell culture and cell lines

HeLa cells (ATCC) were grown in culture flasks in Dulbecco's Modified Eagle Medium (DMEM) (Fisher Scientific) supplemented with 10% (vol/vol) Foetal Bovine Serum (FBS) (Fisher Scientific), 2% l-glutamine (Fisher Scientific), 1% penicillin-streptomycin (Fisher Scientific) and 1% pyruvate (Fisher Scientific) in a humidified static incubator at 5% CO_2_ and 37 °C. When the cells were 70–80% confluent, they were passaged using 0.25% EDTA-Trypsin (Fisher Scientific) as a lifting medium and HBSS (Fisher Scientific) as a wash buffer. De-trypsinised cells were then seeded onto hydrophilised TEM grids at a density of 2 × 10^5^ cells/well and were left for 24 h to adhere on the grids in the incubator (37 °C and 5% CO_2_).

## 7 Method details

### 7.1 Plasmid source

The plasmid pcDNA3.1 Hygro (+) hCx43-EMD was a gift to Professor Agustin Martinez from Professor Viviana Berthoud (The University of Chicago), and it was constructed as follows: the coding region of human Cx43 was PCR amplified with human Cx43 sense and anti-sense primers using human Cx43 DNA subcloned into pcDNA3.1/Hygro(+) (Invitrogen, Carlsbad, CA) as a template and a DNA polymerase. A C-terminal GFP tag was produced by amplification of the Emerald variant (EMD; Invitrogen, Carlsbad, CA) of green fluorescent protein (GFP) using sense and anti-sense primers and Phusion High-Fidelity DNA Polymerase (New England Biolabs, Ipswich, MA). The primers introduced a *Bst*BI restriction site at the end of the coding region of Cx43 and the beginning of the coding region of EMD.

### 7.2 Plasmid transfection

Human Cx43-EMD were transfected into adhered HeLa cells 24 h after seeding in 6-well plates using Lipofectamine 2000 transfection kit (Fisher Scientific), following manufacturer guidelines in a biological safety II laminar flow hood. This involved the pre-incubation of reduced serum media (Opti-MEM) with Lipofectamine 2000, the transfection agent of choice, for 5 min. Thereafter, the Lipofectamine-media mix was added to 2 μg of hCx43-EMD plasmid DNA drop-by-drop and incubated for 20 min at room temperature (Lipofectamine: plasmid ratio = 3:1). The 10% FBS supplemented media which the seeded cells were growing in were replaced with 5% FBS supplemented complete growth media devoid of antibiotics. The homogenised transfection mix was then added to each well containing cells on TEM grids and left for 18–24 h for proper transfection before progressing with the next stages of the experiments. The transfection efficiency was estimated to be between within the region of 30% between 24 and 48 h following transfection (Figs. S2A and B).

### 7.3 Application of pro-inflammatory factors and treatment with a peptide mimetic

Human TNF-alpha recombinant protein (10 ng/mL TNFα; Fisher Scientific), human IL-1 beta recombinant protein (10 ng/mL IL1β; Fisher Scientific) and total media glucose of 32.5 mM (Fisher Scientific) were used to simulate the induction of inflammatory stress in these HeLa cells. Xentry Gap19 (XG19) was added to cells at a final concentration of 5 μM to block Cx43 hemichannels. The exposure of cells to pro-inflammatory factors and treatment with XG19 followed two experimental designs (Fig. 7) detailed below.

Experiment 1 (To determine the protective effect of XG19):

(1) HeLa-parental (non-transfected) cells, then vitrified 24 h after the transfection time point.
(2) HeLa Cx43-EMD transfected cells, then vitrified 24 h after the transfection time point.
(3) Hela-parental (non-transfected) cells, then vitrified 48 h after the transfection time point.
(4) Hela Cx43-EMD transfected cells, then vitrified 48 h after the transfection time point.
(5) HeLa-parental (non-transfected) cells plus pro-inflammatory factors for 24 h and vitrified 48 h after the transfection time point.
(6) HeLa Cx43-EMD transfected cells plus pro-inflammatory factors for 24 h and vitrified 48 h after the transfection time point.
(7) HeLa-parental (non-transfected) cells plus XG19 for 24 h and vitrified 48 h after the transfection time point.
(8) HeLa Cx43EMD transfected cells plus XG19 for 24 h and vitrified 48 h after the transfection time point.
(9) HeLa-parental (non-transfected) cells plus pro-inflammatory factors and XG19 were added together for 24 h and vitrified 48 h after the transfection time point.
(10) HeLa Cx43-EMD transfected cells plus pro-inflammatory factors and XG19 were added together for 24 h and vitrified 48 h after the transfection time point.

Cells with similar conditions as 3–10 above were also vitrified at the 72-h time point post-transfection.

Experiment 2 (To determine the possible curative effect of XG19):

(1) Hela-parental cells, then vitrified 24 h after seeding.
(2) HeLa Cx43-EMD transfected cells, then vitrified 24 h after transfection.
(3) HeLa Cx43-EMD transfected cells plus pro-inflammatory factors for 1 h and vitrified 25 h after transfection.
(4) HeLa Cx43-EMD transfected cells plus pro-inflammatory factors for 1 h, plus XG19 added to cells containing pro-inflammatory factors for an additional 1 h, then vitrified 26 h post-transfection.
(5) HeLa Cx43-EMD transfected cells plus pro-inflammatory factors for 24 h, then vitrified 48 h post-transfection.
(6) HeLa Cx43-EMD transfected cells plus pro-inflammatory factors for 24 h, then the media containing the pro-inflammatory factors were removed, and the transfected cells were left to grow in normal media for an additional 24 h. Vitrification was done 72 h post-transfection.
(7) HeLa Cx43-EMD transfected cells plus pro-inflammatory factors for 24 h, then the media containing the pro-inflammatory factors were removed, and the transfected cells were left to grow in media containing XG19 for an additional 24 h. Vitrification was done 72 h post-transfection.
(8) HeLa Cx43-EMD transfected cells plus pro-inflammatory factors for 45 h. Vitrification was done 72 h post-transfection.
(9) HeLa Cx43-EMD transfected cells plus pro-inflammatory factors for 24 h, then XG19 was added to the media containing pro-inflammatory factors for an additional 24 h. Vitrification was done 72 h post-transfection.

### 7.4 Tracking molecules and organelles with fluorophores

Prior to the vitrification of the samples by plunge freezing, live cell fluorescent dyes were incorporated into the media where the cells were growing. MitoTracker Deep Red (125 nM; Fisher Scientific) for labelling mitochondria, F-Actin CellMask Orange/Red (1 × ; Fisher Scientific) for labelling filamentous actin, LysoTracker Blue (1 μM; Fisher Scientific) for labelling lysosomes and Hoechst 33342 Nuclear Blue (2 drops per mL of media; Fisher Scientific) dyes were all added to each well containing grids and incubated at 5% CO_2_ and 37 °C for 30 min to allow for the cellular internalisation of the dyes.

### 7.5 Preparation of gold nanoparticles

A day before plunge freezing, 1 mL of 250 nm gold nanoparticles (BBI Solutions) was brought out from its stock and left to settle at 4 °C undisturbed. On the day of plunge freezing, 980 μL of the supernatant solution was discarded, and 20 μL of serum-free DMEM was added to the sedimented gold bead pellet. The solution was homogenised and vortexed at speed 5 for at least 30 min to disperse the gold beads.

### 7.6 Addition of gold nanoparticles and plunge freezing

Grids containing adhered cells were mounted onto the Leica GP2 plunge freezer with specialised forceps, and 2 μL of the nanoparticle-DMEM solution was added to the surface of each grid. This was followed by blotting the grids for 2–4 s with Whatman filter paper No.4 to remove excess liquid and reduce the overall thickness of the sample. Each grid was then rapidly plunged into liquid nitrogen-cooled ethane and stored in liquid nitrogen in labelled TEM grid boxes (Agar Scientific) until they were needed for imaging. The grids were constantly maintained in liquid nitrogen to avoid the de-vitrification of the cells.

### 7.7 Fluorescent data collection, reconstruction, and alignment

An AxioImager2 microscope (Carl Zeiss Ltd) coupled to a CMS196 M LED cryo-stage (Linkam) was used to optimise transfection, fluorescent trackers and seeding density. On the cryoSIM microscope (Bespoke made at beamline B24, Diamond Light Source from commercially available parts) [47,50], data were collected using two open-source software packages: Cockpit (Micron, Oxford University) [96] and Python Microscope (Micron, Oxford University). On the cryoSIM workstation, an initial brightfield mosaic was taken to identify areas of interest and ensure the grids had been properly vitrified. Structured illumination data were acquired by exciting the fluorescently labelled cells in pairs with the available four excitation lasers (405 nm-blue, 488 nm-green, 561 nm- orange/red and 647 nm-deep red wavelengths) at 10–50 mW for 10–30 ms, with two channels being collected simultaneously. Structured illumination data was acquired with structured light at 3 angles and 5 phases per Z plane [47,50]. Emitted fluorophores per channel were filtered by the 452 nm (blue), 525 (green), 605 (red), and 655 (deep red) filters and captured by the two cameras (Oxford Instruments) coupled to the cryoSIM. Brightfield images in a Z-stack were also taken for later use during the 3D correlation of cryoSIM and cryoSXT datasets.

Raw structured illumination data were reconstructed using optical transfer function (OTF) files as reference files in SoftWoRX 6.5.2 (GE Healthcare). Chromatic aberration of the different wavelengths was corrected, and data aligned properly with Chromagnon [92].

### 7.8 Cryo soft X-ray tomography and data reconstruction

Cryopreserved samples imaged with the cryoSIM were loaded under high vacuum (10^−8^ Torr) in groups of four into the transmission X-ray microscope (TXM; Carl Zeiss Microscopy, Inc). There, an initial 20 × visible light mosaic was taken of the entire grid to locate regions of interest (ROIs) before a 2D X-ray mosaic of each ROI was captured. Specific 10 × 10 μm areas were chosen for 3D data acquisition within each X-ray mosaic. 3D Soft X-ray data were collected as angular tilt series projections from −60 to +60° at an increment of 0.5° per step with 0.5–1 s exposure on the TXM. At beamline B24, soft X-ray tomography is done using X-ray light at an energy of 500 eV, which lies within the so-called ‘water window’ (the region between the k-absorption edges of carbon at 284 eV and oxygen at 543 eV), where water is transparent, and contrast is generated by the differences in absorption of carbon by different cellular organelles. The data collection strategy on the TXM was carefully thought out to maintain sample integrity and avoid de-vitrification while aiming for the best possible resolution of ∼30 nm.

Tilt series reconstruction into tomograms was done automatically with simultaneous iterative reconstruction technique (SIRT), weighted back projection (WBP) or patch tracking using Batchruntomo [97] based on IMOD [93]. Manual reconstruction of tilt series to tomograms was undertaken using IMOD [93] when the automatic reconstruction was unsuccessful.

### 7.9 Correlation of fluorescence and soft X-ray data

**Fig. 8:**
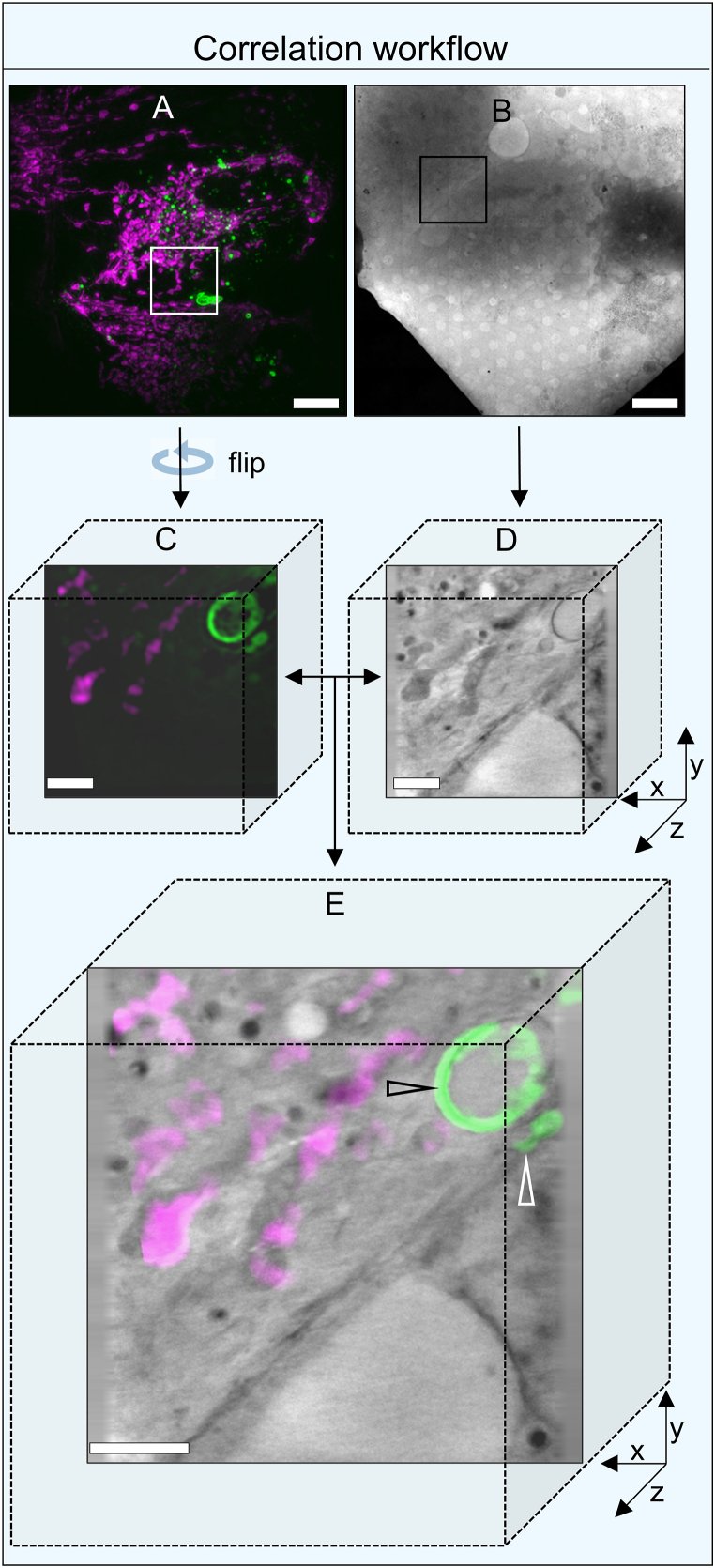
A graphic workflow of the correlative 3D visualisation of connexons and GJs in HeLa cells using fluorescent cryoSIM and soft X-ray tomography at B24. (A) Maximum intensity projection of fluorescent cryoSIM data showing connexin43 in green and mitochondria in magenta. (B) 2D X-ray mosaic of the same area shown in panel A. (C) Slice of 3D-SIM data showing the transformation of fluorescence data to correspond to the orientation and location of the same features seen in the X-ray tomogram (D) Slice of 3D X-ray tomogram of the area in panel B. (E) Correlation of cryoSIM and X-ray dataset from the same area showing the circular loop of internalised connexin43 (connexosome shown with black arrow) and junctional connexin43 (white arrow). Green represents connexin43-EMD tagged connexons, connexosomes or GJ plaques, red represents fluorescently labelled lysosomes, cyan represents fluorescently labelled F-Actin, and magenta represents fluorescently labelled mitochondria. Scale bars (a) 10 μm (b) 10 μm (c–g) 2 μm. (For interpretation of the references to colour in this figure legend, the reader is referred to the Web version of this article.)

Data acquired from both the cryoSIM and TXM were initially visualised and manipulated using Fiji [94]. 2D and 3D point registrations and correlation of both cryoSIM and cryoSXT datasets were achieved using eC-CLEM plugin [95] in Icy (Fig. 8A–E) [98].

## Quantification and statistical analysis

### Data segmentation in Fiji

Data were visualised in Fiji [94], and the mitochondria from the X-ray tomograms were segmented and quantified (surface area and volume) in 3D using the segmentation editor plugin in Fiji. Cell length, cell width, cell aspect ratio, cell circularity and roundness, colocalisation of Cx43 with lysosomes, percentage coverage of Cx43 GJ plaques at cell interfaces, chances for the occurrence of connexosomes at cell interfaces and GJ plaque length were all measured in Fiji from high-resolution fluorescence data captured via cryoSIM.

### Statistical analysis

Results were collated using an Excel spreadsheet (Microsoft), while statistical analyses and data visualisation were executed with Prism 9 (GraphPad). Results were presented as mean ± standard deviation (SD). One-way analysis of variance was used to assess the differences between group means, and the significance level (α) was set at 0.05, where p ≤ 0.05 was statistically significant.

## Data and code availability

Data will be made available on request.

## CRediT authorship contribution statement

**Chidinma Adanna Okolo:** Writing – review & editing, Writing – original draft, Visualization, Supervision, Resources, Project administration, Methodology, Investigation, Funding acquisition, Formal analysis, Data curation, Conceptualization. **Jack Jonathan Maran:** Writing – review & editing, Writing – original draft, Visualization. **Amy Watts:** Writing – review & editing, Writing – original draft, Visualization, Formal analysis. **Jaime Maripillan:** Writing – review & editing, Methodology. **Maria Harkiolaki:** Writing – review & editing, Conceptualization. **Agustín D. Martínez:** Writing – review & editing, Methodology. **Colin R. Green:** Writing – review & editing, Writing – original draft, Conceptualization. **Odunayo Omolola Mugisho:** Writing – review & editing, Writing – original draft, Visualization, Validation, Investigation, Funding acquisition, Conceptualization.

## Declaration of competing interest

The authors declare the following financial interests/personal relationships which may be considered as potential competing interests:Colin R. Green reports a relationship with InflammX that includes: board membership. Colin R. Green reports a relationship with OcuNexus that includes: board membership. Colin R. Green is the founder and Chief Scientific Officer of InflammX Therapeutics which has Xentry-Gap19 as a clinical candidate. Colin R. Green has patent licensed to InflammX Therapeutics. Colin R. Green is a founding scientist of OcuNexus Therapeutics, Inc. (USA) (and previously CoDa Therapeutics, Inc.) which has intellectual property related to the regulation of connexin channels in the treatment of ocular and other diseases. Colin R. Green is part of the inventors on a PCT application regarding Xentry fusion peptides for the modulation of connexin gap junction and hemichannel activity, with this technology licenced by OcuNexus Therapeutics, Inc. (USA). Lola Mugisho has patent licenced to InflammX Therapeutics related to the regulation of connexin channels in the treatment of ocular and other diseases. Chidinma Okolo, Jack Jonathan Maran, Amy Watts, Jaime Maripillan, Maria Harkiolaki and Agustín D Martínez declare no conflict of interest. If there are other authors, they declare that they have no known competing financial interests or personal relationships that could have appeared to influence the work reported in this paper.
