# Supplementary material for: Correlative light and X-ray tomography jointly unveil the critical role of connexin43 channels on inflammation-induced cellular ultrastructural alterations
Source: Heliyon. 2024 Mar 21;10(7):e27888. doi: 10.1016/j.heliyon.2024.e27888 (PMC10979075; doi:10.1016/j.heliyon.2024.e27888)
Supplement: Multimedia component 1 [file mmc1.docx]

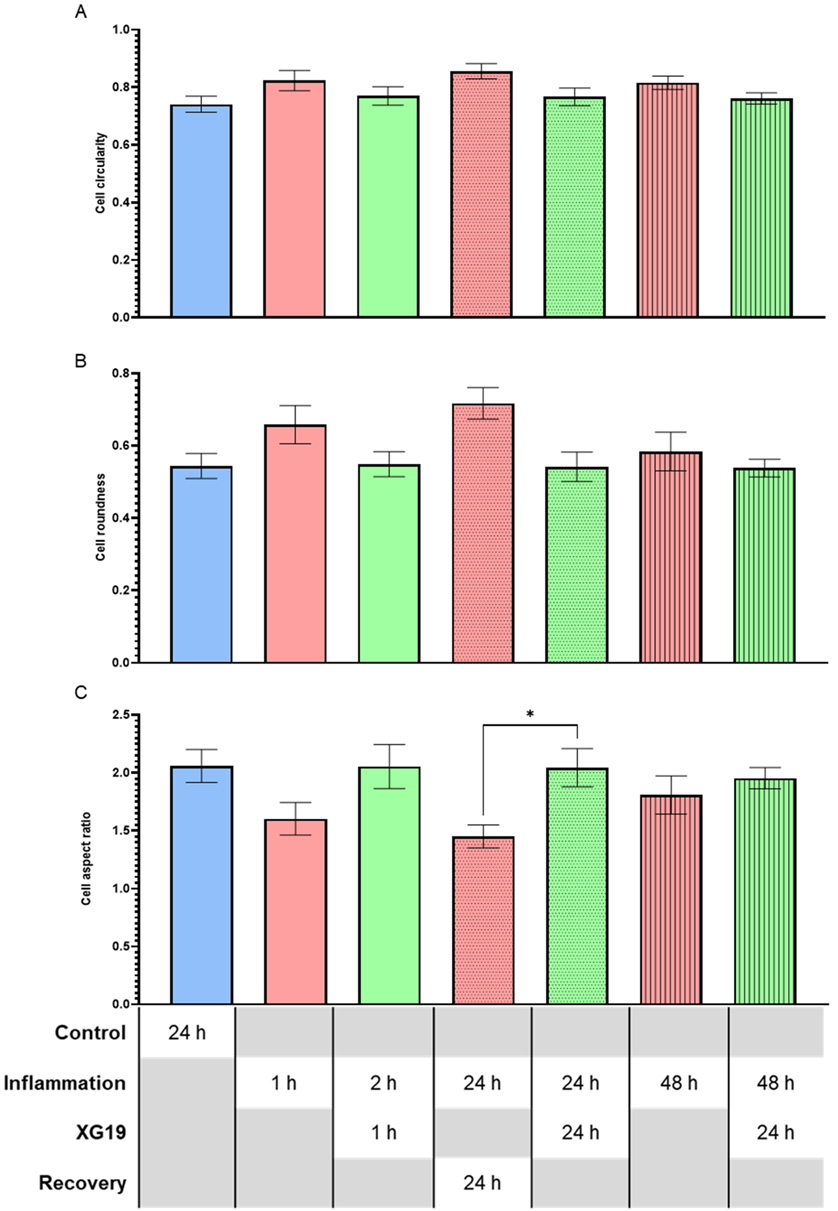


**Figure S1:** Measurements of gross cell morphology as a justification for the need to switch to cell width measurement as a more suited indicator of hypertrophy in these conditions. (A) Cell circularity, (B) Cell roundness and (C) Cell aspect ratio in control Cx43-EMD transfected HeLa cells (blue bar, n = 27), Cx43-EMD transfected HeLa cells at 1 hour following pro-inflammatory factors application (pink bar, n = 8), Cx43-EMD transfected HeLa cells at 2-hours following pro-inflammatory factors exposure plus 1 hour of XG19 co-treatment in the second hour of inflammatory insult (green bar, n = 22), Cx43-EMD transfected HeLa cells at 24 hours of inflammatory conditions + discontinuation of inflammatory trigger for an additional 24 hours (pink dotted bar, n = 10), Cx43-EMD transfected HeLa cells at 24 hours of inflammatory stress + discontinuation of inflammatory stress for 24 hours with 24 hours of XG19 treatment after inflammatory stress was discontinued (green dotted bar, n = 18), Cx43-EMD transfected HeLa cells at 48 hours of exposure to pro-inflammatory factors (pink striped bar, n = 7), Cx43-EMD transfected HeLa cells at 48 hours of exposure to pro-inflammatory factors & 24 hours of XG19 treatment in the last 24 hours under continued inflammatory injury (green striped bar, n = 22). *p < 0.05.


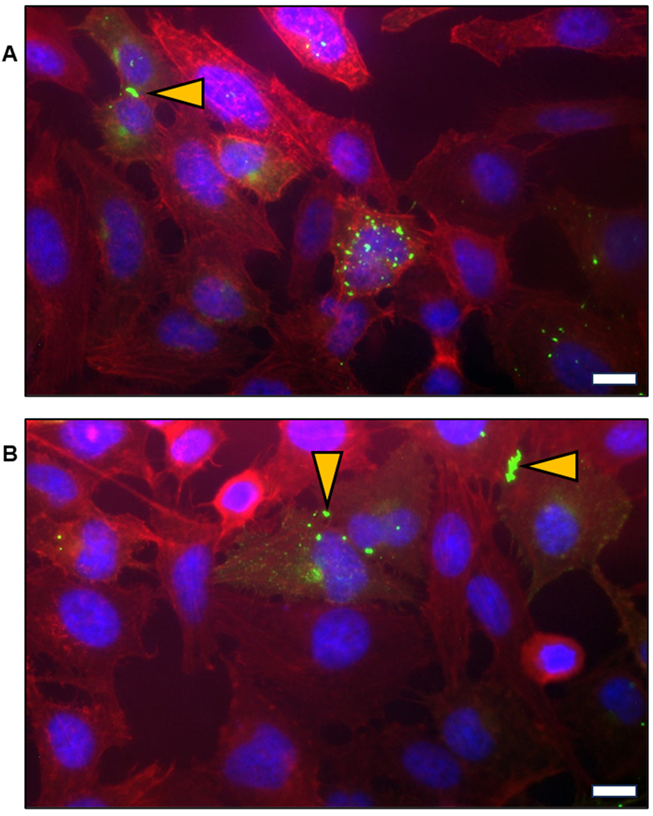


**Figure S2:** An assessment the transfection efficiency of Cx43-EMD in HeLa cells in different regions captured of a glass slide on a conventional fluorescent microscope using 50× objectives. Orange arrows are pointing to gap junction plaques. Scale bars = 30 µm, green represents fluorescent connexin43, red fluorescence represent F-Actin and blue represent DAPI.
